# Novel study on enhancing the ignition pattern of waste and inedible feedstock in a modified diesel engine-enhancing its effectiveness as renewable alternative

**DOI:** 10.1038/s41598-023-45473-w

**Published:** 2023-10-26

**Authors:** K. Rajesh, Chidambaranathan Bibin, M. P. Natarajan, S. Ponnuvel, Yuvarajan Devarajan, T. Raja, Nandagopal Kaliappan

**Affiliations:** 1 Department of Mechanical Engineering, RMK College of Engineering and Technology, Chennai, Tamil Nadu India; 2 Department of Mechanical Engineering, Bharath Institute of Higher Education and Research, Chennai, Tamil Nadu India; 3 Department of Mechanical Engineering, Sri Venkateswara College of Engineering, Chennai, Tamil Nadu India; 4 Department of Mechanical Engineering, Saveetha School of Engineering, SIMATS, Saveetha University, Chennai, Tamil Nadu India; 5 Material Science Lab, Department of Prosthodontics, Saveetha Dental College and Hospitals, SIMATS, Saveetha University, Chennai, Tamilnadu India; 6 Department of Mechanical Engineering, Haramaya Institute of Technology, Haramaya University, Dire Dawa, Ethiopia

**Keywords:** Environmental sciences, Engineering, Mechanical engineering

## Abstract

The objective of the present investigation is to enhance the performance of diesel engine using Capparis spinoza fatty acid distillate biodiesel (CFAB100) at various compression ratios. The experiments were carried out at compression ratios of 16.5:1, 17.5:1, 18.5:1, and 19.5:1. It was noted that an increase in compression ratio from 16.5 to 18.5 resulted in better engine characteristics for CFAB100 and reduced at compression ratio 19.5. Brake-specific fuel consumption of CFAB100 decreased from 0.42 to 0.33 kg/kWh with an increase in compression ratio. The brake thermal efficiency of CFAB100 at a compression ratio of 16.5 is 29.64% lower than diesel, whereas it is 11.32% low at a compression ratio of 18.5. The brake thermal efficiency of CFAB100 is 26.03% higher at a compression ratio of 18.5 compared to 16.5. Due to shorter ignition delay and reduced premixed combustion, the net heat release rate of CFAB100 is lower than diesel at all compression ratios. The peak cylinder pressure for diesel is 56.21 bar, and CFAB100 at compression ratios 16.5, 17.5, 18.5, and 19.5 were 52.36, 55.12, 61.02 and 58.25 bar at full load condition. CFAB100, at a compression ratio of 18.5, had the highest nitrogen oxide emissions (2400 ppm). Carbon monoxide, unburnt hydrocarbon, and smoke showed an average reduction of 46.58%, 40.68%, and 54.89%, respectively, when the compression ratio varied between 16.5 and 19.5. At an optimum compression ratio of 18.5, the CFAB100 resulted in improved performance and emission characteristics that can replace diesel to a possible extent.

## Introduction

Energy plays a predominant role in the growth of a nation, and there is a direct relationship with the level of development^1^. An appropriate energy supply is important for cultural, economic, and social development^2^. Transportation, industry, and households are the important sectors that consume energy and play a major role in creating energy demands^3^. In particular, the transportation sector requires fossil fuels for its energy needs^4^. Following China and the United States, India is the world's third-largest crude oil importer^5^. India imports more than 80% of its crude oil, which has a significant economic impact^6^. Besides the economic side, fossil fuel usage has serious environmental degradation and harmful effects^7^. Researchers anticipate using alternative fuels to meet energy demand in order to preserve the environment and conserve fossil fuel resources^8^. The alternate fuel should be highly efficient and pave the way to sustainable development, environmental preservation, and energy conservation^9^.

The use of diesel engines in automobiles, ships, aircraft, and industrial and agricultural equipment is due to their improved efficiency, better torque, durability, and reliability than gasoline engines^10^. Among various alternative fuels available today, biodiesels are gaining more importance due to their renewable nature, better properties, and eco-friendly nature than exhaustible petroleum fuels^11^. Countries with economies heavily reliant on agriculture, like India, possess ample reserves of biofuels. India is densely populated, and the use of edible oils in biodiesel production is limited due to the crucial requirement for food^12^. Availability and economic aspects of the biodiesel feedstock made the researchers focus on second-generation feedstock (non-edible oils) and third-generation feedstock like fish oil, poultry fat, beef tallow, chicken fats, microalgae, pyrolysis oil, spirulina etc^13^. By-products derived in the process of edible oil refining, namely soap stocks, fatty acid distillates, and acid oil, can be used as a plausible source for biodiesel in the line of third-generation feedstock^14^. The use of the feedstocks mentioned above for biodiesel production is economically viable and leads to sustainability.

Biodiesel and its blends have been studied extensively for their effects on engine performance, combustion, and emissions. Better engine performance was obtained for the biodiesel blend, ranging from 10 to 20%. Numerous efforts have been made to raise the blending percentage of biodiesel with diesel^15^. Several modifications were performed to maintain the engine's performance with increased biodiesel concentration^16^. There were limited studies that specifically addressed the use of 100% biodiesel in compression ignition engines^17^. According to the literature, the utilization of 100% biodiesel necessitates engine modifications to enhance performance and decrease emissions^18^. Injection timing, injection pressure, and compression ratio (CR) are critical engine parameters that substantially impact fuel combustion. The compression ratio has a greater influence on the engine performance, combustion, and emissions characteristics^19^. Biodiesel gains more advantages with an increased compression ratio due to improved fuel properties than neat diesel^20^. Therefore, the effect of varying compression ratios with an increased blending ratio of biodiesel needs to be studied.

Few studies have been conducted on using biodiesel in diesel engines with varying compression ratios. In this view, the effect of tyre pyrolysis oil in a diesel engine with a compression ratio varying from 16.5 to 18.5 and keeping other parameters at the optimum range (like injection pressure and injection timing) was analysed^21^. They reported that the increase in CR from 17.5 to 18.5 resulted in reduced ignition delay, peak cylinder pressure, and higher heat release rate (HRR), which resulted in 8% higher brake thermal efficiency (BTE) and reduced emissions except for NOx. Similar findings with palm biodiesel (B20) when tested with CR 16:1, 17:1, and 18:1 were observed^22^. It was reported that with an increase in CR, a decrease in ignition delay and an increase in peak cylinder pressure. BTE at CR 16:1, 17:1, and 18:1 was found to be 28.9, 30.8 and 33.8%. Hydrocarbon (HC), carbon monoxide (CO), and Smoke opacity showed an average reduction of 47.8, 41.0 and 35.7%. In contrast, a 41.1% increase in NOx. The performance, emission, and combustion of Karanja biodiesel (B100) in a diesel engine with compression ratios varying from 17:1 to 21:1 were evaluated^23^. The result showed that at full load, a BTE increase of 9.5% and 4.6% was observed with engine speeds of 2200 and 3000 rpm at CR 21 than base CR19. The increased CR (21:1) and delayed injection timing improved performance and emissions.

Variable CR with standard IT in a dual-fuel diesel engine was experimented. The results revealed that BTE increased from 16.42 to 20.04% at full load conditions when CR varied from 16:1 to 18:1. With increasing CR, the HC, and CO reduced considerably while NOx and carbon dioxide (CO_2_) were increased to 66.65% and 27.18%. An experimental investigation on the performance characteristics of cardonal-kerosene blends in a VCR engine was conducted^24^. This study involved the testing of these blends at three distinct compression ratios: 16:1, 17:1, and 18:1. The results unveiled a notable improvement in BTE as the compression ratio increased, indicating enhanced engine performance. However, it is noteworthy that an increase in NOx emissions was observed across all the blends, with BK20 (comprising 80% cardonal and 20% kerosene) exhibiting the highest increase, reaching a maximum of 18.7%. The impact of change in compression ratio in DI diesel engines using an ethanol–diesel blend was discussed. Increased compression ratio (17.5:1, 18:5:1 and 19.5:1) and ethanol blend improved performance and combustion with a substantial decrease in emission except for NOx.

The effect of Karanja biodiesel in variable compression multi-fuel engines at compression ratios 15:1, 16:1, 17:1 and 18:1was investigated. It was reported that the BTE for B25 is 5% higher than diesel at full load at CR18. It was concluded that the performance and emission of B25 were better than standard diesel^25^. The effect of biodiesel derived from waste cooking oil at CR 15:1 and 17.5:1 was experimentally investigated. They tested biodiesel blends of B20, B40, B60, B80 and B100 at both CR and compared them with standard diesel. B40 blend showed higher BTE at CR 17.5:1 at full load. It was observed that with an increase in load, the BSEC decreased at both CR 15:1 and 175:1, whereas CO emission is higher at CR 15:1 than at 17.5:1. Experiments were performed using castor oil in the VCR engine to study the combustion characteristics^26^. The biodiesel was blended with diesel up to 50%, and the compression ratio varied from 15:1 to 18:1. Mean gas temperature (MGT) and cylinder pressure increased with increasing compression ratio for all the blends. In contrast, the net heat release (NHR) was observed to be reduced with an increase in CR.

The effect of waste fried oil methyl ester (WFOME) in a VCR diesel engine was investigated. The test was carried out with blends of B50 and B70 at different compression ratios of 14.5, 16.5 and 17.5. It was noted that the biodiesel blends showed performance close to diesel and improved emission characteristics^27^. They concluded that WFOME showed reduced BSFC and emissions with increased brake power and BTE with increasing compression ratio. The impact of compression ratio on combustion and performance characteristics of DI diesel engines was investigated. They tested the engine at CR 16:1, 17:1 and 18:1 for standard diesel fuel. The results showed that BSFC and EGT reduced from CR 16 to 18. A rise in peak pressure for increasing compression ratio and a maximum rate of pressure rise of 5.38 bar/℃ was observed at CR18 at full load.

According to the literature review, compression ratio and biodiesel have a substantial impact on the performance, combustion, and emission characteristics of diesel engines. Several studies were conducted in a diesel engine with fixed compression ratios for various biodiesel and biodiesel-diesel blends. Furthermore, few studies have been conducted on the influence of compression ratio on higher biodiesel mixes, and very few works have been conducted utilizing neat biodiesel. Very limited works were reported using CFAB as fuel in diesel engines. Thus, the direct use of neat CFAB becomes an important area of research in existing diesel engines with minor modifications. Use of neat biodiesel blended with diesel to overcome viscosity and poor atomization, which adversely affect the engine performance. All of these difficulties have prompted us to seek a new environmentally favorable renewable fuel that can be employed in existing diesel engines.

### Motivation and objective of the present investigation

To meet the energy requirements of the transportation sector, countries like India greatly depend on oil imports. Due to drawbacks associated with conventional fuels, more focus was given to fuel derived from renewable sources. India is a leading vegetable oil-producing country next to the United States of America, China, and Brazil. Capparis spinoza oil is obtained from the Capparis spinoza seeds. In 2019–20, next to the Philippines and Indonesia, India was the third-largest producer of Capparis spinoza oil and produced 4,75,800 tonnes. To improve the oil's usefulness, Capparis spinoza oil refining removes impurities, such as insoluble solids, free fatty acids, moisture, gums, and other compounds. CFAD and refined Capparis spinoza oil were produced at the end of the physical refining of crude Capparis spinoza oil.

CFAD is produced at the last refining stage, during deacidification/deodorization, and maintained in a vacuum. During this stage, steam at 220–240 °C is passed, and the output of the stage is refined Capparis spinoza oil and CFAD. The CFAD derived during the refining process can be used as an alternative source for producing biodiesel. The biodiesel obtained from CFAD is the least expensive than edible-grade vegetable oils. Based on the extensive literature review, few works were published discussing the effect of performance, combustion, and emission characteristics of biodiesel derived from third-generation feedstocks under varying compression ratios. Limited investigations were presented on the performance characteristics of biodiesel derived from the by-products of vegetable oil refining. The present investigation tests CFAB100 (100% Capparis spinoza fatty acid distillate biodiesel) in a direct injection diesel engine at various compression ratios to determine its performance, combustion, and emission characteristics.

## Materials and methods

### Fuel preparation from CFAD

This study complies with relevant institutional, national, and international guidelines and legislation. The CFAD is filtered to remove suspended impurities and heated to 100–120 ℃ to eliminate moisture. It was determined that the CFAD had an acid value of 49 mg KOH/g. The feedstock’s greater acid value necessitates utilizing a two-step process for synthesizing biodiesel: esterification and transesterification. The first esterification process was carried out with the following conditions: molar ratio 10:1, H_2_SO_4_ catalyst concentration 2.5%, temperature 60 ℃, and reaction time 90 min. The methanol and H_2_SO_4_ were added when the oil reached 60 °C, and the reaction was continued for 90 min. The ester formed was obtained by phase separation, and the CFAD ester’s acid value is 3.6 mg KOH/g (FFA 1.8%), suitable for transesterification. Transesterification of ester was performed with a molar ratio of 8:1, KOH catalyst of 0.5%, reaction temperature of 50 ℃, reaction time of 40 min and agitation speed of 600 rpm. A similar reaction procedure of esterification was followed for transesterification to obtain biodiesel. The mixture at the end of the reaction is transferred to a separation funnel and allowed to sit for 24 h. The extracted raw biodiesel, which consists of unreacted catalysts, methanol, soap, etc., was rinsed with distilled water. The water used for rinsing is eliminated by heating the biodiesel to obtain neat biodiesel. The physicochemical properties of the CFAD, CFAB100 and diesel were measured according to ASTM methods and are in Table. 1.

**Table 1:** Physicochemical properties of CFAD, CFAB100 and diesel.

Properties	CFAD	CFAB100	Diesel
Kinematic Viscosity @40 °C (cSt)	10.34	4.30	2.3
Density @15 °C (kg/m^3^)	892	879	810
Flash Point (°C)	175	90	55
Calorific Value (kJ/kg)	37,790	36,069	42,500
Saponification value	249.54	221.1	–
Iodine value (g Iodine/100 g)	8.66	9.5	
Cloud point (°C)	1	− 1	
Pour point (°C)	0.6	− 3	− 6
Acid value (mg KOH/g)	49	1.6	–
Free fatty acid (% as Lauric)	24.5	0.8	–
Oxidation stability (h at 110 °C)	6.5	7.6	30.6
Cetane Number	56.3	61	46–51

### Characterisation of CFAD oil and its biodiesel

#### GC analysis of Capparis spinoza fatty acid distillate

**Figure 1:**
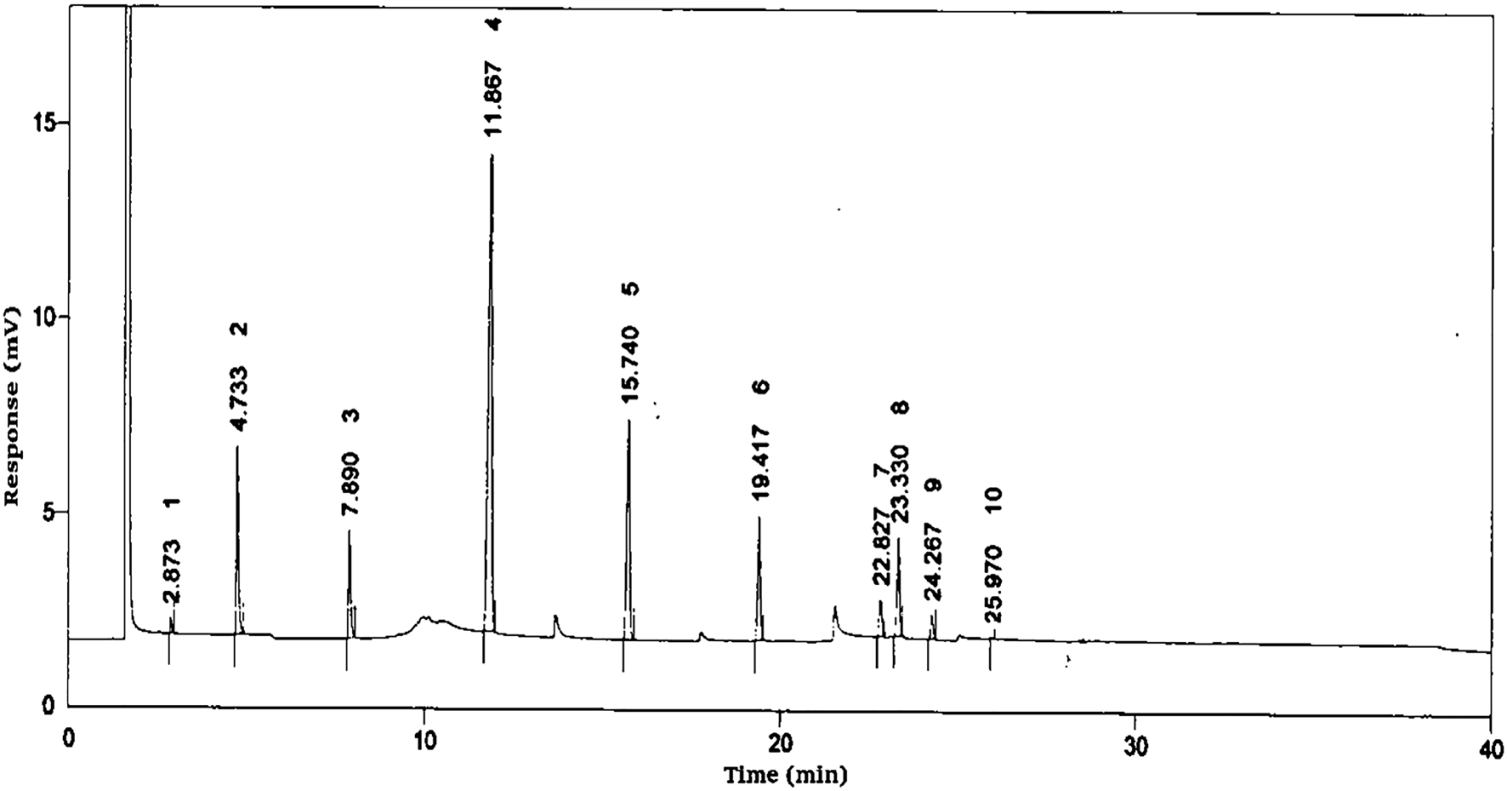
Gas chromatography analysis of CFAD.

The fatty acid content of biodiesel feedstock considerably impacts the fuel's performance, combustion, and emission characteristics. The composition of various fatty acids present in the CFAD was determined using Gas chromatography analysis. The GC analysis revealed the presence of medium-chain saturated fats such as Caproic acid (0.72%), Caprylic acid (10.21%), Capric acid (6.75%), Lauric acid (45.53%), Myristic acid (16.92%), Palmitic acid (8.84%), Stearic acid (2.51%) and unsaturated fats such as Oleic acid (6.78%), Linoleic acid (1.63%), Linolenic acid (0.06%). Thus, 91.53% of the CFAD comprises saturated fats, whereas only 8.47% are unsaturated fats. The peak distributions of CFAD recorded during the GC test are shown in Fig. 1.

#### FTIR analysis of Capparis spinoza fatty acid distillate biodiesel

**Figure 2:**
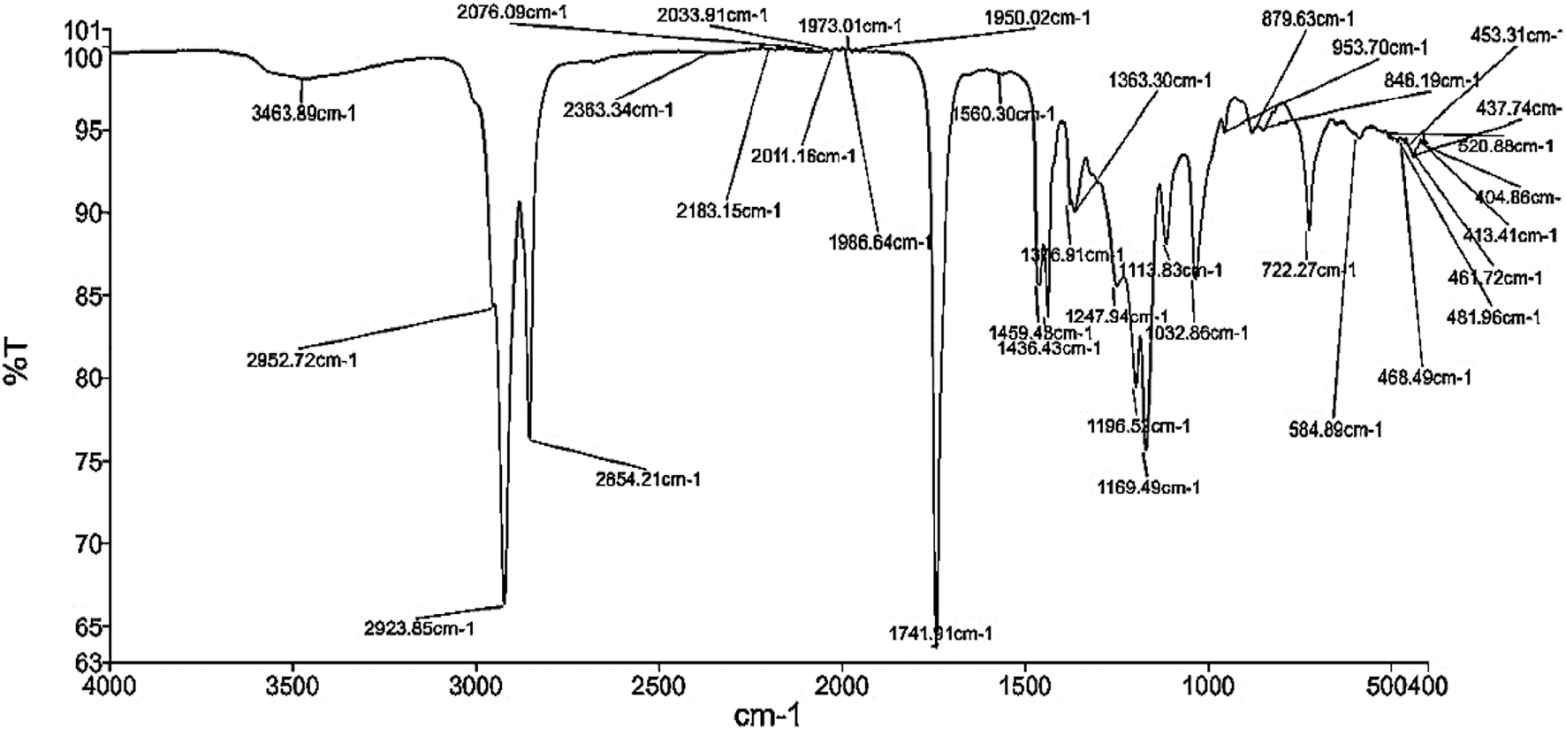
FTIR spectra of Capparis spinoza fatty acid distillate biodiesel.

Functional groups in CFAB100 were determined using Fourier transform infrared spectroscopy (FTIR). The FTIR (Spectrum Two FT-IR/Sp10 Software, PERKIN ELMER, USA) analysis was performed on CFAB100 between the spectral regions 400–4000/cm as per the ASTM E1252. From Fig. 2, it is clear that there is no presence of water or alcohol in the biodiesel since there is no peak in the wavelength region 3100–3500/cm. C–H stretch vibration (alkane functional group) is observed from the peak in the wavelength of 2923.85/cm and 2854.21/cm. Wavelengths between 1720 and 1760/cm show a peak stretching of the carbonyl group. The peak 1741.91/cm represents the presence of esters, which is common in the region 1700–1800/cm. The strong vibration stretches observed between 1470 and 1350/cm show an alkane functional group and bending vibrations. The peak present between the wavelength regions 900–1300/cm is caused by alcohol functional groups with C–O stretching. Further, the area between 650 and 900/cm indicates the presence of an alkane functional group with C–H stretch bending vibrations.

### Experimentation

**Figure 3:**
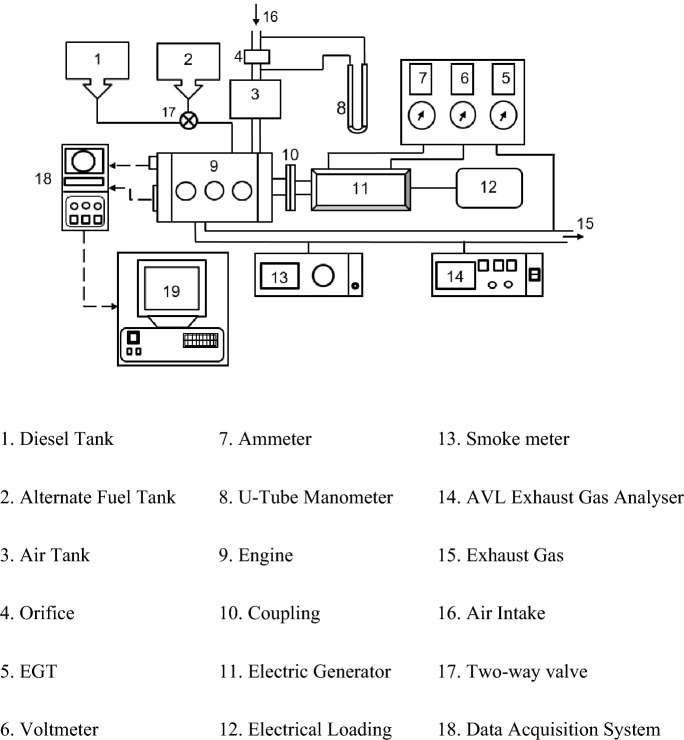
A schematic of the diesel engine used for the study.

**Figure 4:**
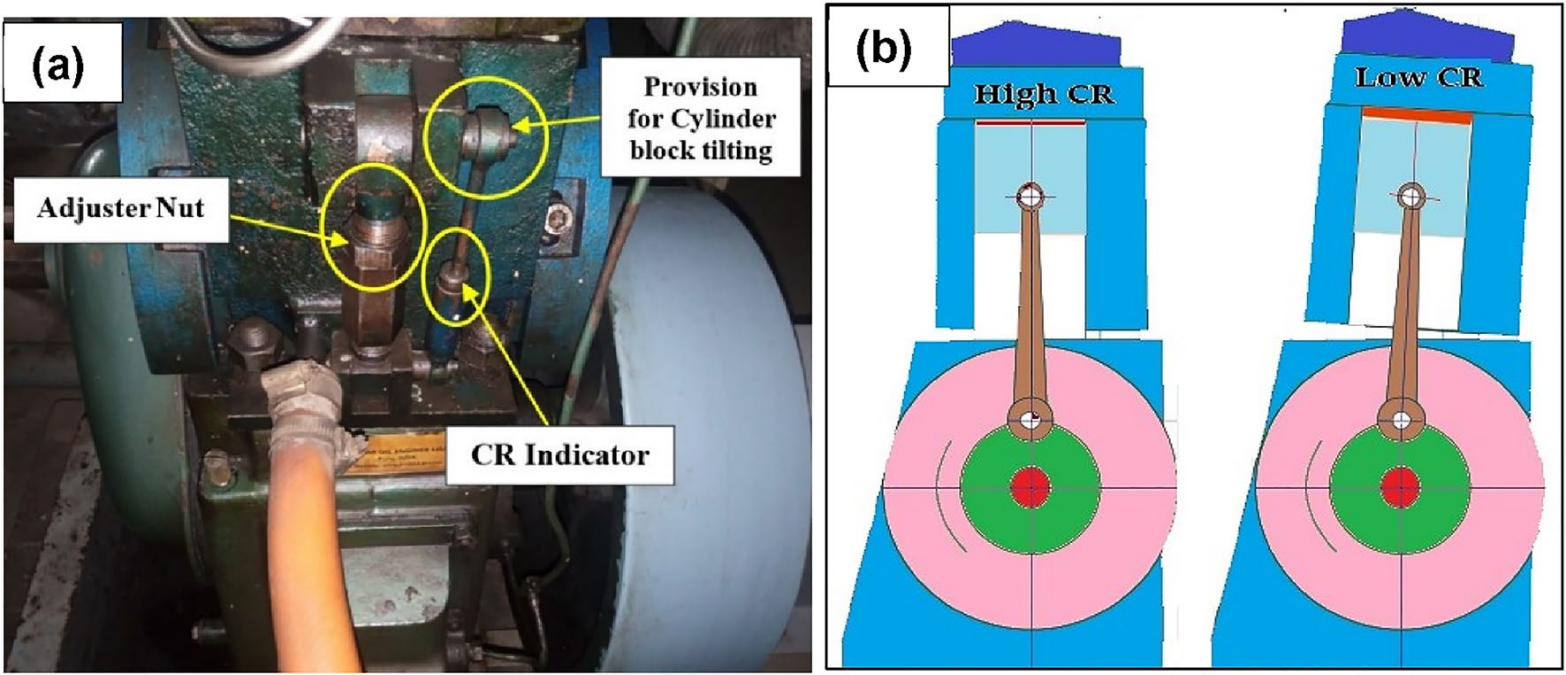
(**a**) Compression ratio adjusting mechanism. (**b**) Tilting block arrangement showing the variation of CR.

Kirloskar’s VCR single-cylinder, four-stroke water-cooled variable compression ratio engine is utilized in this investigation. Figure 3 shows the schematic representation of the experimental diesel engine configuration used for the study. Using the tilting cylinder block design shown in Fig. 4, the modification in the CR was carried out without affecting the geometry of the combustion chamber. The geometry specification details of the experimental engine are given in Table 2. An eddy current dynamometer was utilized to load the engine at various speeds. ‘Enginesoft,’ an analyzing software, is used for online performance evaluation. Using an AVL DIGAS 444N five gas analyzer, the CO, UHC, and NOx emissions from the tailpipe were measured. A Diesel tune digital smoke head, model DX260 smoke head, was used to determine the smoke opacity.

**Table 2:** Experimental diesel engine specification.

Engine parameters	Specifications	
Model		Kirloskar TV1
Engine type		Single-cylinder, 4-stroke, vertical diesel engine
Cooling system		Water cooled
Maximum power		3.5 kW @ 1500 rpm
Compression ratio		12:1–22:1
Cylinder bore		87.5 mm
Stroke length		110 mm
Connecting rod length		234 mm
Dynamometer		Eddy current, water-cooled
Injection timing		23° before TDC
Injection pressure		200 bar
Software		Engine performance analysis software “Enginesoft.”

The engine’s steady-state condition was achieved by allowing it to run for nearly 20–30 min under no-load conditions. The experiments were carried out once the oil and cooling water temperatures were stabilized. The engine was tested with neat diesel and 100% biodiesel (CFAB100) under varying load conditions. The experiments were repeated at various compression ratios CR16.5, CR17.5, CR 18.5, and CR19.5 at the rated speed of 1500 r.p.m. The experimental test run for the diesel fuel was performed at CR 17.5 to create baseline data to compare with CFAB100 at various CRs. The uncertainty analysis was performed to evidence the accuracy of the experiment. The linearized approximation approach of uncertainty was used to compute the percentage uncertainty of various parameters. Table 3 shows the accuracy and uncertainty values for various parameters. The calculation of the overall experimental uncertainty was performed by the formula as shown below.

$${\text{Overall}}\;{\text{ uncertainty}} = \sqrt {\sum \left( {Uncertainty{ }\;of{ }\;each{ }\;parameter} \right)^{2} }$$

$$\begin{aligned} {\text{Overall }}\;{\text{uncertainty}}\; & \, = \;{\text{ square }}\;{\text{root}}\;{\text{ of }}\left[ {\left( {{\text{engine}}\;{\text{ load}}} \right)^{{2}} } \right. + \, \left( {{\text{engine}}\;{\text{ speed}}} \right)^{{2}} + \, \left( {{\text{time}}} \right)^{{2}} \\ & \;\;\; + \, \left( {{\text{exhaust }}\;{\text{temperature}}} \right)^{{2}} + \, \left( {{\text{Engine }}\;{\text{power}}} \right)^{{2}} + \, \left( {{\text{fuel }}\;{\text{flow}}} \right)^{{2}} + \, \left( {{\text{BTE}}} \right)^{{2}} + \, \left( {{\text{BSEC}}} \right)^{{2}} \\ & \;\;\; + \left( {{\text{HC}}} \right)^{{2}} + \, \left( {{\text{CO}}} \right)^{{2}} + \, \left( {{\text{NOx}}} \right)^{{2}} + \, \left( {{\text{Smoke }}\;{\text{opacity}}} \right)^{{2}} \\ & = {\text{ Square }}\;{\text{root}}\;{\text{ of }}\left[ {\left( {0.{2}} \right)^{{2}} + } \right.\left( {{1}.0} \right)^{{2}} + \left( {0.{2}} \right)^{{2}} + \left( {0.{1}} \right)^{{2}} + \left( {{1}.0} \right)^{{2}} + \left( {{1}.0} \right)^{{2}} + \left( {{1}.0} \right)^{{2}} \\ & \;\;\; + \left( {{1}.{5}} \right)^{{2}} + \left( {0.{2}} \right)^{{2}} + \left( {0.{2}} \right)^{{2}} + \left( {0.{2}} \right)^{{2}} + \left( {{1}.0} \right)^{{2}} \\ & = \, \pm { 2}.{73}\% \\ \end{aligned}$$

**Table 3:** Various instruments used and their uncertainties.

Measurement	Instrument	Accuracy	Uncertainty (%)
Engine load	Strain gauge type load cell	± 0.1 kg	± 0.2
Speed	Speed sensor	± 10 rpm	± 1
Time	Digital stopwatch	± 0.2 s	± 0.2
Exhaust temperature	K-type thermocouple	± 1 ℃	± 0.1
Fuel flow	DP transmitter	–	± 1
Engine power	–	–	± 1
BSEC	–	–	± 1.5
BTE	–	–	± 1
UHC	AVL Gas analyser	± 15 ppm	± 0.2
CO	AVL Gas analyser	± 0.02%	± 0.2
NOx	AVL Gas analyser	± 20 ppm	± 0.2
Smoke opacity	Smoke meter	± 0.1	± 1

## Results and discussion

### Engine performance

#### Brake thermal efficiency

**Figure 5:**
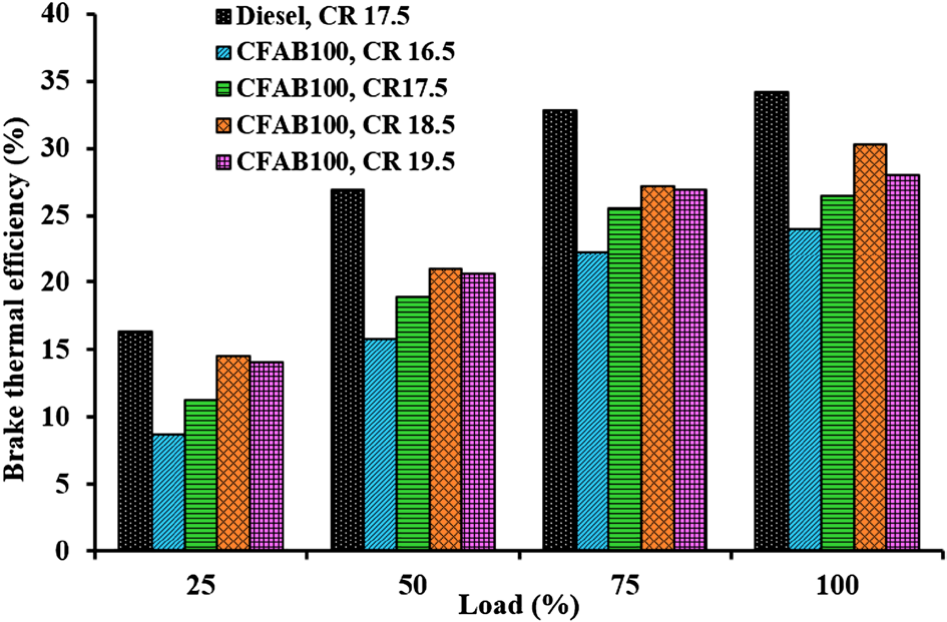
Variation of BTE concerning load.

Figure 5 depicts the change in brake thermal efficiency versus load at various CRs for diesel and CFAB100. The results showed that BTE increased for both test fuels with an increase in load. This is due to an increase in load resulting in more brake power, enhanced combustion temperature and reduced losses at higher loads^28^. The BTE obtained for diesel at CR 17.5 were 16.37, 26.87, 32.86, and 34.17 concerning load 25, 50, 75 and 100%, respectively. Diesel has a greater BTE value than CFAB100 at all engine loads and all compression ratios. This is because diesel has a larger calorific value, a low viscosity, and a low fire point than diesel. The effect of varying the compression ratio can be seen from the increase in engine efficiency at higher compression ratios. At full load, the BTE for CFAB100 at CRs 16.5, 17.5, 18.5 and 19.5 were found to be 24.04%, 26.25%, 30.30% and 28.03%. Lower calorific value, increased density or viscosity, poor atomisation and vaporisation of CFAB100 may result in a lower BTE at lower CR. On the other hand, higher air temperature in the cylinder due to a higher compression ratio signified improved air–fuel mixing and rapid evaporation. As a result, the ignition delay is reduced, hence enhanced combustion near TDC, improving the BTE. It is clear from the net heat release rate (Fig. 10) the peak attained is very close to TDC, which implies that the heat release during expansion stroke is reduced, which arises due to late combustion. Also, the biodiesel’s better lubricity nature helps reduce frictional losses. The BTE at CR 19.5 is reduced compared to CR18.5. This may be attributed to fuel spray impingement on the piston due to reduced clearance volume at higher CR^29^. This results in improper air–fuel mixing, which results in reduced BTE. However, the BTE of CFAB100 significantly improved by 26.03% at CR 18.5 compared to CR 16.5.

#### Brake-specific fuel consumption

**Figure 6:**
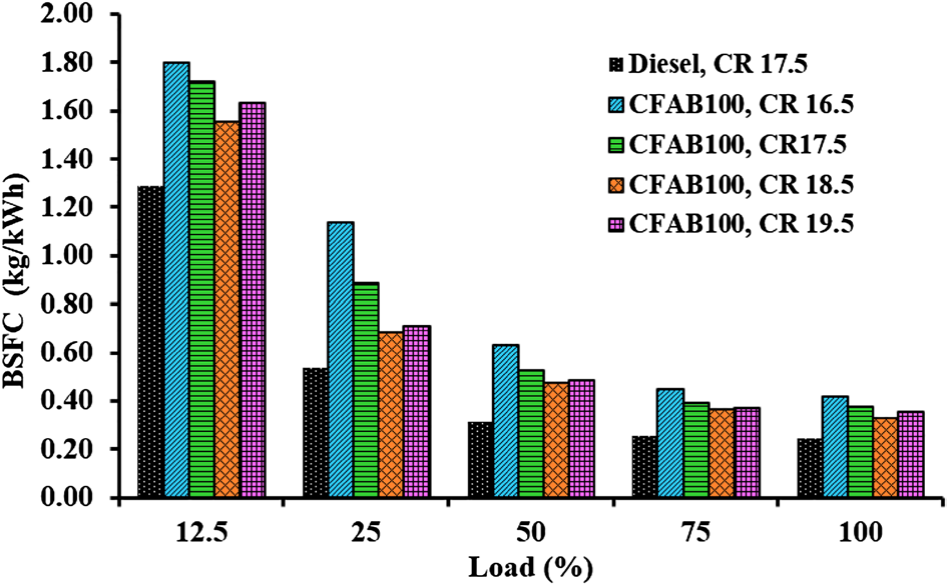
Variation of BSFC concerning load.

Figure 6 shows the relationship between engine load and brake-specific fuel consumption for diesel and CFAB100 at various compression ratios. In the present study, When the engine load is increased, the BSFC for diesel and CFAB100 exhibits a declining trend for all compression ratios. The combustion process deteriorated with lower engine loads, resulting in lower in-cylinder temperatures and CFAB oil molecules burning with reduced velocity. It was noted that the BSFC of CFAB100 is higher compared to diesel. Due to biodiesel's lower calorific value and higher viscosity, more fuel has to be put into the engine to get the same amount of power^30^. The BSFC values of diesel and CFAB100 at CRs 16.5, 17.5, 18.5 and 19.5 were 0.25 kg/kWh, 0.42 kg/kWh, 0.38 kg/kWh, 0.33 kg/kWh and 0.36 kg/kWh. Figure 6 shows a decrease in CFAB100's specific fuel consumption as the compression ratio increases.

At CR 16.5, the BSFC is 40.47% higher than diesel; at CR 18.5, it is only 24.24%. The reduced combustion chamber area and clearance volume variation at higher CR contribute to the improved air intake and concise fuel admission, resulting in better combustion and reduced heat loss. Combustion chamber temperature rises as the compression ratio increases, reducing the disadvantages arising from high viscosity and poor volatility of CFAB100, which may cause poor combustion, thereby decreasing BSFC.

#### Exhaust gas temperature

**Figure 7:**
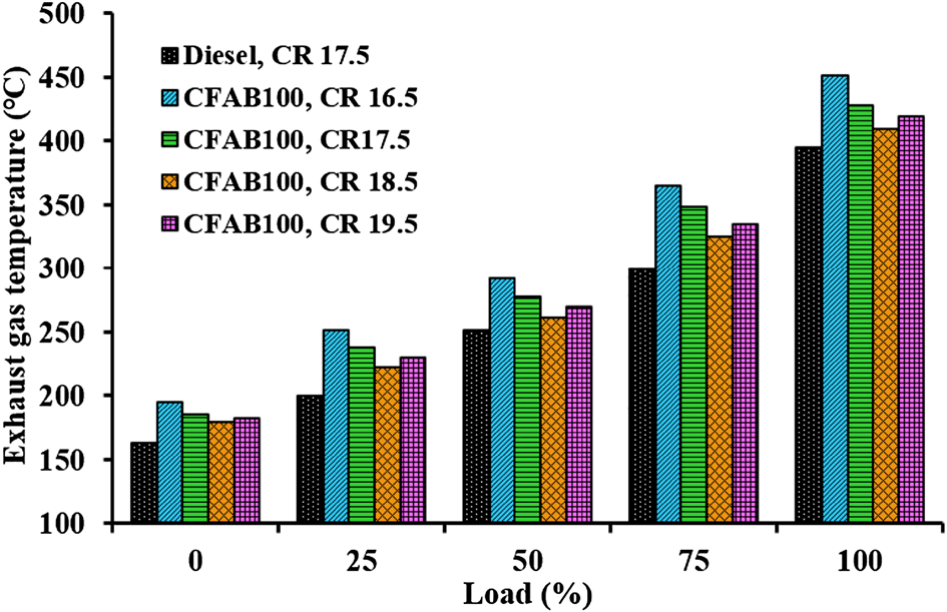
Variation of EGT concerning load.

The impact of the compression ratio on diesel and CFAB100 on the exhaust gas temperature (EGT) under various engine loads is illustrated in Fig. 7. The EGT is related to fuel combustion quality and the efficient utilisation of heat energy. The EGT provides a clear understanding of the air–fuel ratio, oxygen availability, the heat released during the diffusion combustion phase and engine performance.

At higher loads, more fuel must be injected into the combustion chamber to satisfy the increased power demands, which causes an increase in EGT at all compression ratios. It is noted that the EGT is higher for CFAB100 at CR 16.5, 17.5, 18.5 and 19.5 than diesel. The EGT varies from 163 to 395 °C at CR 17.5 for diesel. The EGT of CFAB100 varies from 195 to 451 °C at CR 16.5, 185–428 °C at CR 17.5, 180–410 °C at CR 18.5, and 183–421 °C at CR 19.5. The increasing trend is due to a decrease in the thermal efficiency of CFAB100, resulting in increased heat loss in the exhaust and a higher fuel consumption rate. The biodiesel fuel particles continue to burn in diffusive combustion due to higher cetane numbers and poor volatility. Higher EGTs at low CR indicate the engine is thermally overloaded. The enhanced burning of the fuel that occurs at higher compression ratios leads to a decrease in the EGT of CFAB100 to a greater extent. Reduced ignition delay in response to elevated air temperature favoured enhanced fuel burning at higher CR, thus lowering the fuel quantity available for diffusive combustion^31^.

### Combustion analysis

#### Variation of cylinder pressure with crank angle

**Figure 8:**
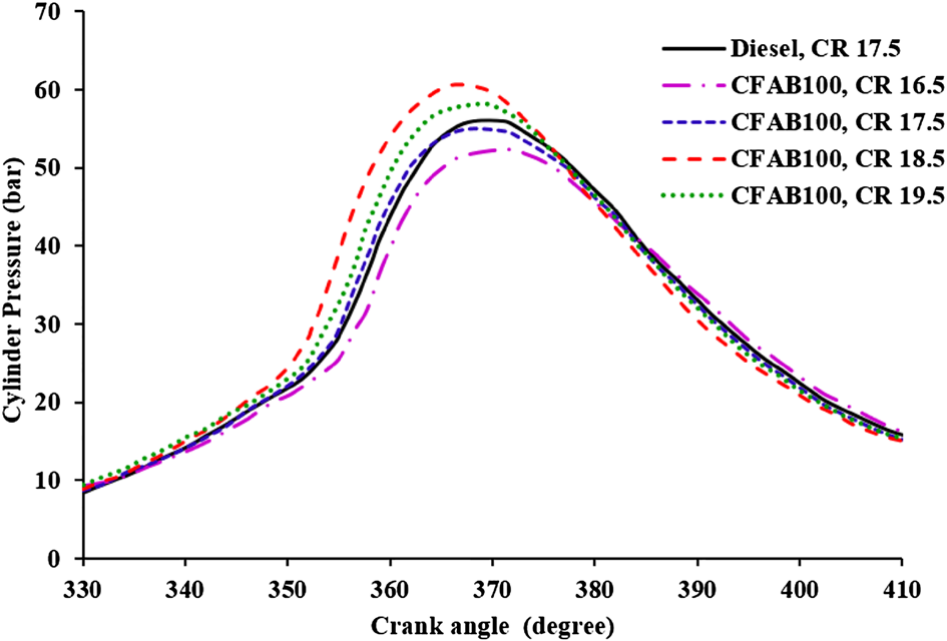
Variation of cylinder pressure with crank angle.

**Figure 9:**
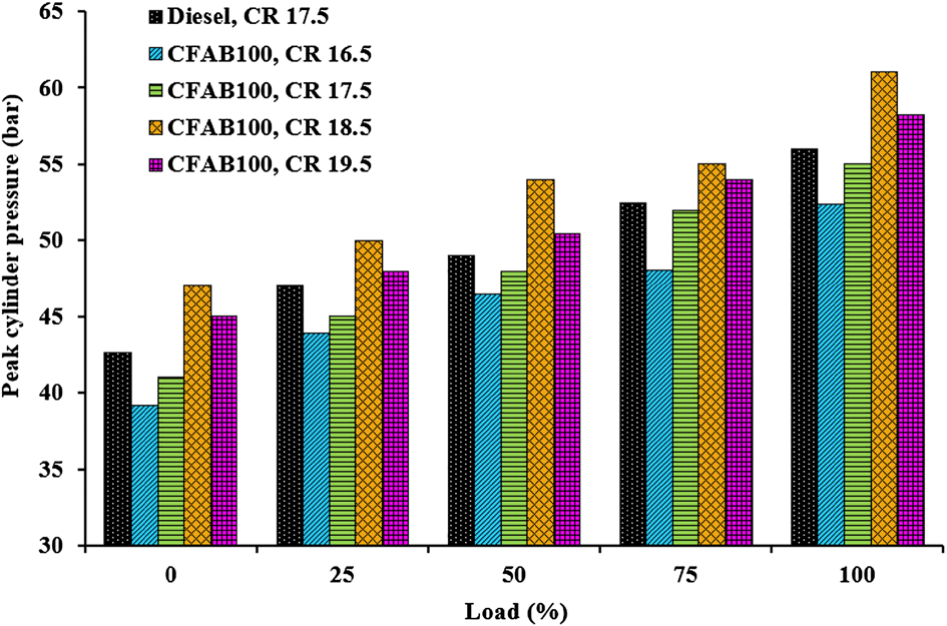
Variation of peak cylinder pressure with load.

Figure 8 illustrates diesel and biodiesel cylinder pressure at varied crank angles at full load conditions at different CRs. The percentage of fuel burned in the premixed combustion phase determines the maximum cylinder pressure in diesel engines^32^. Figure 9 indicates that as load increases, cylinder pressure rises due to a large volume of fuel injected into the cylinder. The combustion of biodiesel starts earlier due to reduced ignition delay than diesel, which is clear from Fig. 8. Figure 9 shows that the peak pressure of CFAB100 reduces with the decrease in the CR. A decrease in peak pressure at lower CR could be due to the lower calorific value, low volatility, high kinematic viscosity and density of CFAB100, which can affect the spray pattern and fuel evaporation, thereby decreasing cylinder peak pressure. Also, the inappropriate mixing of burned and unburned fuel mixtures and poor swirl resulted from slow combustion reducing peak pressure at lower CR. The increase in CR has a greater impact on biodiesel than diesel. Maximum pressure is noted for CFAB100 (61.02 bar) at CR 18.5. This is attributed due to complete combustion favoured by (1) higher cetane number, which reduces the fuel accumulation during the ignition delay period, (2) time available for combustion is more due to reduced ignition delay, (3) the presence of oxygen in the biodiesel contributes to an improvement in the combustion process and (4) better fuel atomization and vaporization at higher CR^33^.

#### Net heat release rate (NHRR)

**Figure 10:**
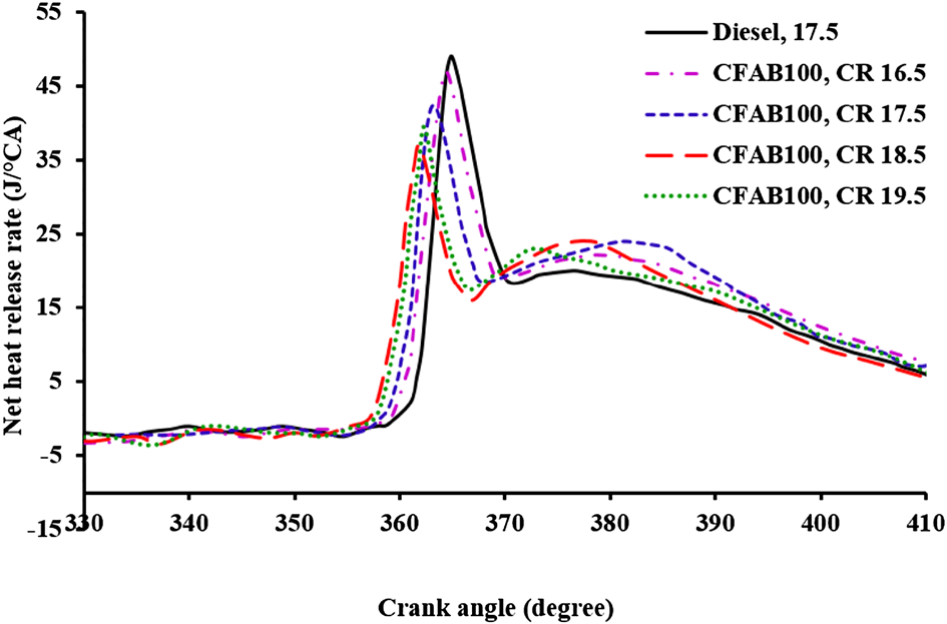
Variation of net heat release rate with crank angle.

Figure 10 shows the NHRR for the test fuels at different CR under full load conditions. Negative NHRR is observed for both test fuels due to the cooling effect produced due to the evaporation of the injected fuel at the end of compression before the start of combustion^19^. The NHRR rises gradually and moves towards positive after the start of combustion. The NHRR of diesel is found to be higher compared to CFAB100 at all CRs. Longer ignition delay, which provides more time for better mixing of incoming charge and higher calorific value of diesel, results in high NHRR. From Fig. 10, the NHRR of CFAB100 is shifted significantly to the left due to its higher cetane number, which promotes an early ignition of the fuel. The increase in compression ratio has an inverse effect on the NHRR for CFAB100, which can be noted from the values 47.41 J/°CA, 42.71 J/°CA, 37.38 J/°CA and 39.52 J/°CA for CR 16.5, 17.5, 18.5 and 19.5. Higher NHRR is observed at lower CR due to poor combustion caused by i) high viscosity and surface tension, which affects the fuel atomization, and ii) poor volatility and high latent heat, increasing the time for vaporization. On the other hand, an increase in CR favours high temperature inside the cylinder, which is responsible for viscosity reduction, better fuel spray formation and the advanced start of combustion^34^. This is responsible for burning a portion of the premixed fuel during the ignition delay, and the remaining fuel takes part in the premixed combustion leading to reduced NHRR.

#### Cumulative heat release (CHR)

**Figure 11:**
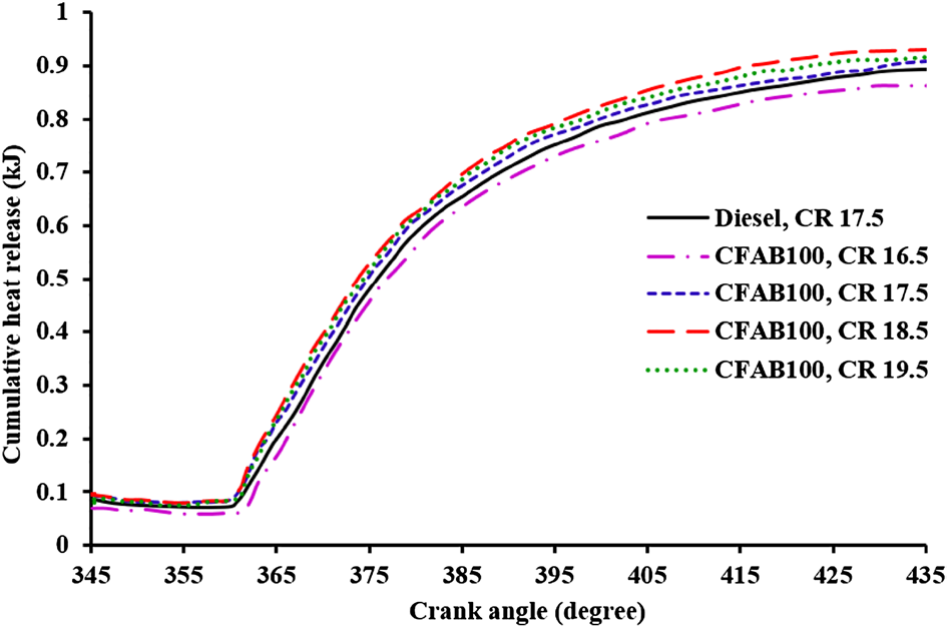
Variation of cumulative heat release with crank angle.

Figure 11 depicts the relationship between the crank angle and the variation in the cumulative heat release of diesel and CFAB100. The cumulative heat release is calculated based on the net cumulative heat release and the energy supplied by the fuel per cycle^35^. At the start of combustion, the CHR decreases due to the heat absorbed for fuel vaporization during the ignition delay period to form a combustible mixture. It is noted from Fig. 11 the CHR of the test fuels attained a maximum value after the start of combustion and followed a similar trend. At full load conditions, the CHR of diesel is 0.892 kJ, and CFAB100 at CRs 16.5, 17.5, 18.5 and 19.5 were 0.848 kJ, 0.908 kJ, 0.929 kJ and 0.912 kJ. The CHR of CFAB100 at CR18.5 is higher than diesel and CR 16.5, 17.5 and 19.5. The higher value is attributed to the higher air density, cylinder pressure and temperature, significantly improving the overall combustion process. Also, at a higher CR, the duration of premixed combustion is reduced due to the early start of fuel combustion^36^. At the same time, the amount of heat lost to the surroundings is reduced compared to diffused combustion phase, which results in higher CHR^37^. The CHR of CFAB100 at CR18.5 is increased by 3.5% compared to diesel.

### Engine emissions

#### Carbon monoxide

**Figure 12:**
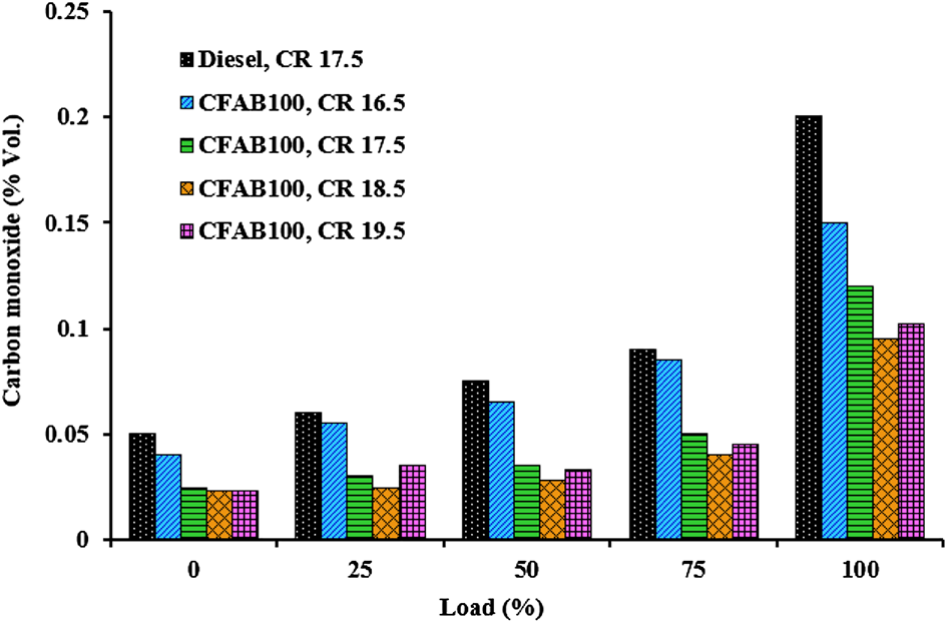
Variation of carbon monoxide with load.

Figure 12 depicts the relationship between carbon monoxide emissions and engine load for diesel and CFAB100. The rate of CO formation depends on the fuel decomposition and oxidation rate, which is influenced by the air–fuel mixture temperature and availability of the unburnt fuel^38^. The characteristics of the fuel and the amount of time necessary for its complete combustion are the factors that influence the generation of carbon monoxide. Higher CO is observed with increased load due to more fuel injected at higher loads. It can be seen from Fig. 12. the diesel CO is higher than CFAB100. CFAB100 consists of a higher percentage (92%) of shorter-chain saturated fatty acids, which reduce the boiling point and improve carbon monoxide's oxidation rate. CFAB fuel molecules have inherent oxygen, which improves combustion efficiency and thus reduces CO emissions compared to diesel^39^.

When the compression ratio is increased, it is observed that the quantity of CO emission produced by CFAB100 decreases. It is observed that the CO emission of CFAB100 at CR 19.5 is slightly higher than at CR 18.5. This may be due to fuel spray impingement on the piston due to the reduced clearance volume of the combustion chamber^40^. This creates a fuel-rich region that tends to increase the CO formation at CR 19.5 than CR18.5. The average decrease in CO% of CFAB100 than diesel at CR 16.5, 17.5, 18.5 and 19.5 were 16.84%, 41.26%, 50% and 46.51%. Higher combustion temperature prevails inside the chamber, creating a suitable environment for easy mixture oxidation at a higher compression ratio. It is already discussed that an increase in compression ratio reduces ignition lag, providing more time for diffusion combustion^41^. Thus, the total combustion process is improved, resulting in lower CO emissions.

#### Unburnt hydrocarbon

**Figure 13:**
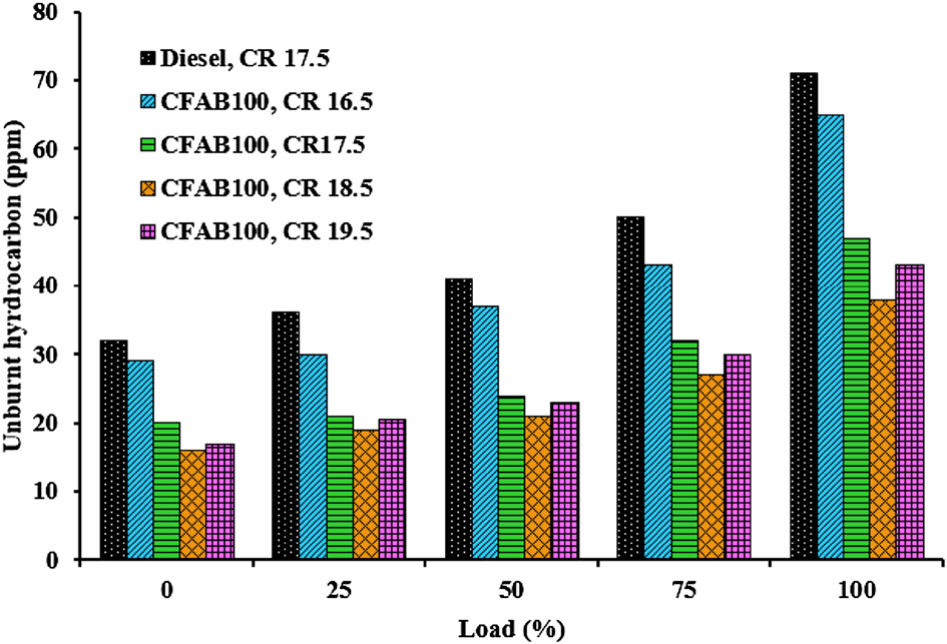
Variation of unburnt hydrocarbon with load.

Figure 13. depicts the variation of unburnt hydrocarbon with engine load at different compression ratios. The characteristics of unburnt hydrocarbons depend on air–fuel ratio, fuel structure, fuel spray characteristics, and engine operating conditions. The UHC emission nearly follows a linear trend concerning an increase in load. Reduced UHC emission is observed at low loads compared to higher loads due to increased combustion duration. More fuel injected per cycle at higher load conditions leads to a rich mixture in the local area, substantially increasing UHC. The highest UHC emission is observed for diesel at all load conditions. The higher cetane number of biodiesel due to its high saturated fatty acid content leads to reduced ignition delay, and a higher equivalence ratio helps oxidize the unburnt hydrocarbons^42^. It is clear from Fig. 13. the UHC decreases with an increase in the compression ratio for CFAB100. At 100% load, the UHC values of diesel and CFAB100 at CR 16.5, 17.5, 18.5 and 19.5 are 71 ppm, 65 ppm, 47 ppm, 38 ppm and 43 ppm, respectively. The temperature increase at the end of the compression stroke contributes to enhanced combustion. Apart from that, reduced ignition delays due to the absence of aromatic HC and sulphur in CFAB100 favours a quick start of combustion. This gives the mixture of air and fuel sufficient time to burn, which leads to better combustion. At CR 19.5, the UHC of CFAB100 showed a slight increase due to the fuel layer on the top surface of the piston. The high surface tension of CFAB100 affects the air–fuel mixture formation, leading to unburnt fuel particles^43^. When CR is raised from 16.5 to 18.5, it results in a 20% average reduction in UHC compared to diesel.

#### Nitrogen oxides

**Figure 14:**
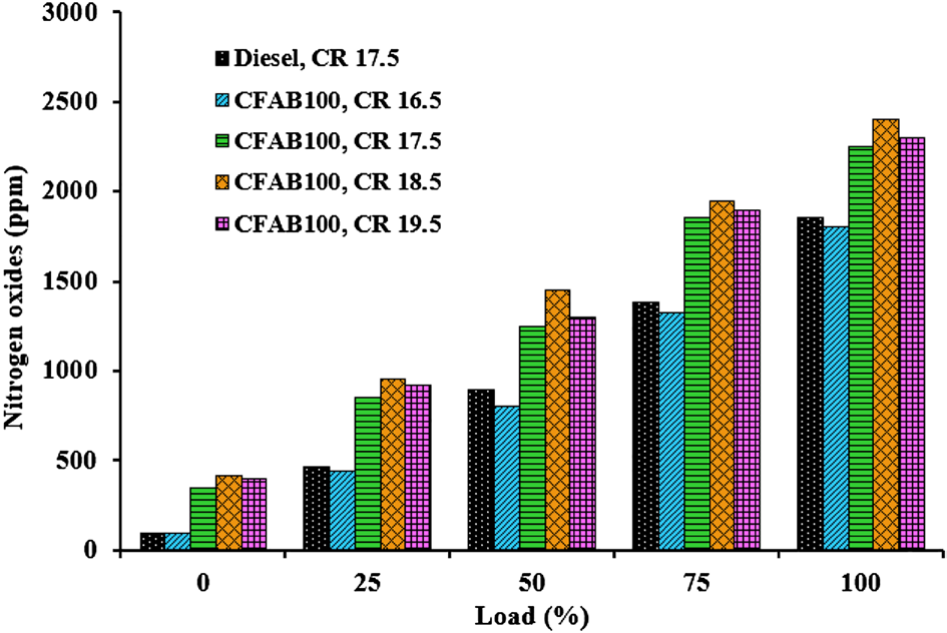
Variation of nitrogen oxides with load.

The NOx emission of diesel and CFAB100 at different compression ratios are plotted against engine load given in Fig. 14. When biodiesel is used as fuel, factors that have a greater effect on NOx emission are physicochemical properties of the biodiesel, adiabatic flame temperature, rich oxygen content, biodiesel molecular structure, premixed burn fraction, chemical kinetics effect, ignition delay time, ignition timing and engine loading condition**.** It is clear from Fig. 14 that NOx produced by CFAB100 is greater than diesel in all conditions. The main causes for this trend: (1) higher adiabatic temperature of biodiesel due to its oxygenated nature results in better combustion, which leads to increased temperature and NOx emission^44^; (2) Reduced ignition delay favours longer residence time for the air–fuel mixture and primary combustion products owing to higher NOx^36^; (3) Fuel composition has a variable effect on NOx formation based on the degree of unsaturation and chain length. NOx tends to increase for CFAB100 because its composition has a high degree of shorter chain-length fatty acids^45^. The NOx emission, on average, increased by 25.01% for CFAB100 when the CR varied from 16.5 to 18.5, respectively. Increased NOx emission is seen with increasing CRs at all loads. The significant factor supporting the NOx formation is the higher combustion temperature, which increases with increased CR. Also, better combustion is observed for biodiesel at higher CR, resulting in reduced soot formation. Soot formed in the flame zone is highly responsible for radiative heat transfer. Therefore, a decrease in soot formation reduces the heat transfer, thereby maintaining bulk flame temperature, thus leading to higher combustion chamber temperature and formation of thermal NOx^46^.

#### Smoke opacity

**Figure 15:**
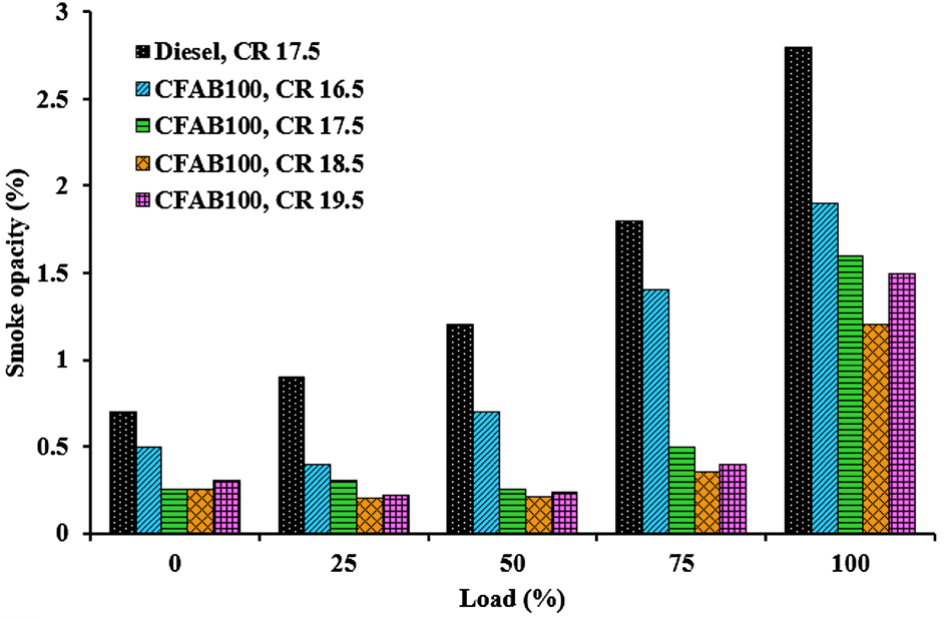
Variation of smoke opacity with load.

Figure 15 reveals the impact of CR on the smoke opacity of CFAB100 compared with diesel under different engine loads. The smoke emission from the engine comprises unevenly shaped, clustered tiny carbon particles formed due to the unavailability of oxygen^47^. Incomplete combustion occurs when additional fuel is provided to sustain conditional loading, leading to a rise in smoke emissions as the load increases. The biodiesel at different compression ratios produced lesser smoke, as seen in Fig. 15. Due to the biodiesel's increased oxygen content, a leaner air–fuel mixture is formed^48^. This supports the oxidation of carbon to carbon monoxide and carbon dioxide instead of participating in the reactions of soot formation. Also, the methyl ester fuel has no aromatic contents, reducing the formation of solid carbon (soot). Smoke emission decreases by 30% on average at CR 18.5 compared to CR 16.5; this indicates that increasing the compression ratio reduces smoke emission. Clearance volume reduces as the compression ratio increases, causing the temperature and pressure in the combustion chamber to increase. This favors better fuel atomization, evaporation and quick combustion resulting in better combustion, thereby reducing smoke emission^49^.

## Conclusion

In the present study performance, the VCR diesel engine’s combustion and exhaust emission characteristics under varying loading conditions with diesel and CFAB100 were discussed. Improvement in engine characteristics was observed for CFAB100 when CR varied from 16.5 to 18.5 and declined on further increasing the CR to 19.5. The conclusions derived based on the test results are given below,

- The CFAB100’s BTE improved as the compression ratio increased. BTE of CFAB100 at CR 18.5 is 30.30%, which is 26.03% higher compared to CR 16.5 at full load. At the same time, CFAB100 at CR18.5 is 11.32% less than diesel at CR17.5.
- CFAB100 has a higher BSFC than diesel because of its lower heating value. The highest BSFC value is recorded for CFAB100 at CR 16.5 (1.80 kg/kWh), whereas diesel at CR17.5 showed a lower value (1.29 kg/kWh) Average reduction of 29.59% BSFC is observed when CR varied from 16.5 to 18.5 for CFAB100.
- It was observed that the EGT of the CFAB100 is greater under all operation conditions than diesel. The maximum temperatures are observed for CFAB100 at CR 16.5 because of the burning of biodiesel fuel particles during controlled combustion.
- The combustion of biodiesel fuel started earlier, with an increase in CR from 16.5 to 19.5. The in-cylinder pressure at CR18.5 is maximum due to shorter ignition delay, high in-cylinder temperature, and burning of more fuel in premixed combustion.
- NHRR values lowered due to enhanced in-cylinder temperature and pressure corresponding to higher CRs. The maximum NHRR values obtained were 47.41 J/°CA, 42.71 J/°CA, 37.38 J/°CA and 39.52 J/°CA for CR 16.5, 17.5, 18.5 and 19.5 respectively.
- The CO, UHC, and smoke opacity showed an average reduction of 51.58%, 47.39%, and 60.89% when CR varied from 16.5 to 18.5 with CFAB100 against that of diesel. However, the average increase in NOx is 34.32%.

From the above results, it is concluded that increasing the compression ratio has improved the performance, combustion, and emission characteristics of Capparis spinoza fatty acid distillate biodiesel and is comparable with diesel. To use CFAB100 as fuel for a diesel engine, it is advisable to choose a compression ratio of 18.5 for improved performance and emission characteristics.

## Data Availability

The datasets used and/or analysed during the current study available from the corresponding author on reasonable request.
